# Extracts of Qizhu decoction inhibit hepatitis and hepatocellular carcinoma *in vitro* and in C57BL/6 mice by suppressing NF-κB signaling

**DOI:** 10.1038/s41598-018-38391-9

**Published:** 2019-02-05

**Authors:** Ling-feng Wan, Jian-jiang Shen, Yao-hui Wang, Wei Zhao, Nan-yuan Fang, Xin Yuan, Bo-yu Xue

**Affiliations:** 1 Department of Infectious Disease, Affiliated Hospital of Nanjing University of Chinese Medicine, 155 HanZhong Road, Nanjing, 210029 Jiangsu China; 2 Department of Laboratory, Affiliated Hospital of Nanjing University of Chinese Medicine, 155 HanZhong Road, Nanjing, 210029 Jiangsu China; 3 School of Laboratory Medicine, Chengdu medical college, 783 Xindu Road, Chengdu, 610500 Sichuan China; 4 The First Clinical Medical College, Nanjing University of Chinese Medicine, 155 HanZhong Road, Nanjing, 210029 Jiangsu China

## Abstract

Hepatitis and hepatocellular carcinoma are serious human diseases. Here, we examined the *in vivo* and *in vitro* inhibitory effect of extracts of Qizhu decoction (a traditional Chinese medicine) on hepatitis caused by diethylnitrosamine or hepatitis B virus and on diethylnitrosamine-induced hepatocellular carcinoma. The results showed that both *the aqueous and ethanol extracts (QC and QS, respectively)* of Qizhu decoction significantly inhibited hepatic inflammation and liver cancer induced by diethylnitrosamine or hepatitis B virus by suppressing NF-κB signaling and decreasing the levels of TNF-α and IL-1β. Both QC and QS inhibited the proliferation and migration of primary cancer hepatocytes by reducing cyclin B1, cyclin D1 and N-cadherin expression and increasing E-cadherin expression. QC and QS also promoted the apoptosis of primary cancer hepatocytes by upregulating caspase-3 and downregulating BCL-2 expression. The knockdown of p65 in NF-κB signaling inhibited the ability of QC and QS to significantly reduce the colony formation ability of liver cancer cells. Additionally, QC and QS might significantly inhibit the DNA replication of hepatitis B virus *in vivo* and *in vitro*, and we found that corilagin and polydatin were the active compounds of QC and QS. Taken together, our *in vitro* findings and our results in C57BL/6 mice showed that extracts of Qizhu decoction might inhibit hepatitis and hepatocellular carcinoma by suppressing NF-κB signaling.

## Introduction

Inflammation is an integral component of the hepatic wound-healing response to injury induced by hepatitis viruses, excess dietary fat, cholestasis, alcohol and other carcinogens^1,2^ and might be strongly linked to the development of fibrosis, cirrhosis and hepatocellular carcinoma (HCC)^2–5^. Most HCCs occur in patients with hepatic fibrosis or cirrhosis, and the chronic wound-healing process (or inflammation) in the liver is an essential driver of hepatocarcinogenesis^1^. HCC is the second leading cause of cancer-related deaths worldwide and has an incidence of approximately 850,000 new cases per year^6^. HCC represents approximately 90% of all cases of primary liver cancer, and there is currently no highly effective drug or therapy available for the treatment or cure of this deadly disease^7^. Chronic hepatitis B virus (HBV) infection accounts for more than half of all HCC cases^8^. It has been estimated that more than 275 million individuals are chronically infected with HBV and face a 15–40% lifetime risk of developing end-stage liver disease, including cirrhosis, liver failure and HCC^9–12^. Thus, a better understanding of the pathogenesis of HCC is necessary to develop better treatments.

It has been reported that the inflammation-related NF-κB pathway plays an important role in liver cancer^13^. Furthermore, NF-κB has a wide range of functions in different cellular compartments, and these include influencing the survival of hepatocytes, inflammation in Kupffer cells, and the survival, inflammation and activation of HSCs^1^. In mouse models, the genetic ablation of NF-κB regulators also leads to spontaneous liver injury, fibrosis and HCC^14,15^.

Traditional herbal medicines are attracting increasing amounts of attention due to their potential for the treatment of a variety of diseases. In this study, we focused on extracts from Qizhu decoction, a formula used in traditional Chinese medicine. Qizhu decoction consists of several herbal components, namely *Phyllanthus urinaria*, *Polygonum cuspidatum*, *Hedyotis diffusa*, *Paeonia lactiflora*, *Paeonia veitchii*, *Rhizoma Smilacis Glabrae*, and *Salvia miltiorrhiza*^16^. Furthermore, this decoction has been used to promote blood circulation and particularly to enhance the body’s immunity^17^ and also facilitates the restoration of thymus atrophy and hypofunction caused by malnutrition. Moreover, it has been reported that compounds or extracts from *P. urinaria* and *P. cuspidatum* exhibit antioxidant activity^17^, inhibit HBV-induced hepatitis^18–20^, and show activity against CCl_4_-induced liver injury and schistosomiasis-induced hepatic fibrosis^16,21^. However, the effects of extracts of Qizhu decoction on HCC are not well understood. To determine whether Qizhu decoction can be used in the clinic, we first investigated the therapeutic effects of Qizhu decoction using animal experiments and then explored the potential mechanisms of Qizhu decoction against liver disease *in vitro*. In this study, we found that extracts from Qizhu decoction might inhibit both hepatitis caused by diethylnitrosamine (DEN) or HBV and DEN-induced liver cancer by suppressing NF-κB activity.

## Materials and Methods

### Preparation of extracts

We first prepared extracts of Qizhu decoction (provided by Nanjing University of Traditional Chinese Medicine) as follows: the plants were ground and extracted with either double-distilled water or 70% ethanol according to previously described method^16,22^. Both the aqueous extract (QC) and the 70% ethanol extract (QS) were then dried to a powder, dissolved in phosphate buffered saline (PBS) to obtain a concentration of 100 mg/mL, and stored at −20 °C. The chemical compositions of QC and QS were determined by liquid chromatography/quadrupole time-of-flight mass spectrometry (LC-QTOF-MS) at Nanjing University of Traditional Chinese Medicine. The reference standards, polydatin, corilagin, ethanol and all the other reagents were purchased from Sigma-Aldrich (St. Louis, MO, USA).

### Cell culture and transfection

HepG2 and PLC/PRF/5 cells were obtained from the American Type Culture Collection (ATCC, MD, USA). The cells were grown at 37 °C and 5% CO_2_ in Dulbecco’s modified Eagle’s medium (DMEM) (Gibco, CA, USA) supplemented with 10% fetal bovine serum (Gibco), 100 mg/L streptomycin and 0.1 U/L ampicillin. Twenty-four hours prior to transfection, 7 × 10^5^ HepG2 cells were seeded onto six-well plates and incubated overnight. When the cells reached 60% confluency, they were transfected with 4 μg of pHBV1.1 using Lipofectamine 2000 (Invitrogen, Carlsbad, CA, USA) diluted with serum-free DMEM according to the manufacturer’s instructions.

The small interfering RNAs (siRNAs) used for the siRNA experiments were synthesized by GenePharma (Shanghai, China). The p65 siRNA sequence was 5′-GATCAATGGCTACACAGGA-3′, and the negative control siRNA sequence was 5′-TTCTCCGAACGTGTCACGT-3′. The siRNAs were transfected into primary cancer hepatocytes or other cells using Lipofectamine 2000 (Invitrogen). Forty-eight hours after transfection, the cells were harvested to obtain the total RNA or protein samples.

### Animals

Male C57BL/6 mice (aged 6 weeks) were purchased from Nanjing University, China, and housed in a standard vivarium with 12-h light/12-h dark cycles and free access to food and water. The study was approved by the Animal Care and Protection Committee of Nanjing University-Gulou Hospital (SYXK 2004-0013). The authors confirmed that all the animals received humane care, and all the animal experiments were performed in accordance with the relevant guidelines and regulations. The protocol for generating mice with DEN-induced hepatitis and HCC was modified from a previously described method^2^. Briefly, according to the protocol, DEN-treated mice were injected intraperitoneally (i.p.) once per day with 50 mg/kg body weight DEN (Sigma-Aldrich) dissolved in normal saline. After one week of DEN administration, the Qizhu decoction extracts (20 g/kg body weight p.o.) were administered daily until the end of the experiment (28 weeks). Similarly, each control animal was given a single i.p. injection of normal saline and then administered corn oil by oral gavage three times per week.

HBV-transgenic mice were administered the Qizhu decoction extracts (20 g/kg body weight p.o.) daily until the end of the experiment. The HBV-transgenic mouse line C57BL/6J-TgN (Alb1 HBV) 44 Bri, which expresses part of the HBV genome, including the S, pre-S, and X genes, under the mouse albumin promoter, was purchased from the Jackson Laboratory (Bar Harbor, ME, USA). These HBV-transgenic mice and their wild-type littermates, which were sex- and age (6 to 8 weeks)-matched, were used in the experiments^23,24^. In addition, Guangzhou brown spotted ducks with congenital and acquired duck hepatitis B virus infection were purchased from a hatchery (Chaoyang Village, Guangzhou, China). These ducks were housed in a standard vivarium with 12-h light/12-h dark cycles and free access to food and water.

### Isolation of primary cancer hepatocytes

Mouse primary cancer hepatocytes were isolated as previously described^25^. The mice were anesthetized with 1% sodium pentobarbital (Amresco, USA) and dissected. Their hepatic portal veins were washed for blood collection, and collagenase (Sigma-Aldrich) perfusion was performed. The livers were immediately moved to a sterile 10-cm cell culture dish for mincing, and the hepatocytes were then dispersed by aspiration with a large-bore pipette, filtered through a 70-μm membrane (Millipore, Shanghai, China) to remove any tissue debris, washed twice with cold DMEM, and centrifuged at 50 *g* and 4 °C for 4 min. The isolated hepatocytes were seeded in 6-cm dishes at a density of 1 × 10^7^ cells/dish in DMEM with 10% fetal bovine serum (FBS), and 6 h after seeding, the medium was changed to new DMEM (Invitrogen) containing 10% FBS (Invitrogen).

### Histology

The livers were excised and fixed in 10% formalin buffer. The fixed specimens were embedded in paraffin blocks, sectioned, and stained with hematoxylin and eosin (H&E).

### Measurement of TNF-α and IL-1β production

The effects of the Qizhu extracts (QC and QS) on the production of TNF-α and IL-1β were measured using ELISA kits according to the manufacturer’s instructions (R&D Systems, Inc., Minneapolis, MN, USA). Briefly, the isolated primary cancer hepatocytes, HepG2 cells, and PLC/PRF/5 cells (1 × 10^5^ cells/mL) were plated in 24-well plates and pretreated with the indicated concentrations of QC and QS, and culture medium supernatants (100 μL) were collected for ELISAs^26^. The DEN-induced liver cancer tissue was lysed and centrifuged, and the supernatant was collected for ELISAs according to previous reports^26^.

### Isolation of total RNA, reverse transcription polymerase chain reaction (RT-PCR), and quantitative PCR (qPCR)

The TRIzol reagent was purchased from Invitrogen, the RT-PCR reagents were purchased from Promega (Madison, WI, USA), and the qPCR reagents were purchased from Bio-Rad. Total RNA was isolated using the TRIzol reagent according to the manufacturer’s instructions. RNA (1 μg) was reverse transcribed using M-MLV reverse transcriptase to produce cDNA. qPCR was performed using a Mastercycler with specific primers, and glyceraldehyde-3-phosphate dehydrogenase (GAPDH) was used as an internal control. The primers (GenScript Co., Ltd., Nanjing, China) used in the study are the following: TNF-α, 5′-GCG ACG TGG AAC TGG CAG AAG-3′ (forward) and 5′-TCC ATG CCG TTG GCC AGG AGG-3′ (reverse); IL-1β, 5′-TCT CAT TGT CTC GGT GCT C-3′ (forward) and 5′-CTT TCG GGA AGA GGT TTC A-3′ (reverse); and GAPDH, 5′-CAC CAT CTT CCA GGA GCG AG-3′ (forward) and 5′-GCA GGA GGC ATT GCT GAT-3′ (reverse). The relative levels of the mRNAs were then determined through the 2^−ΔΔCt^ method using GAPDH as the internal control.

### Western blot analysis

After treatment with the Qizhu decoction extracts, the tissues or cells were analyzed by immunoblotting. Samples of total protein was obtained by gently lysing the cells for 40 min with lysis buffer (20 mM sucrose, 1 mM EDTA, 20 μM Tris-Cl pH 7.2, 1 mM DTT, 10 mM KCl 1.5 mM MgCl_2_, 5 μg/mL pepstatin A, 10 μg/mL leupeptin, and 2 μg/mL aprotinin), and supernatants were collected after centrifugation. For western blot analysis, an equal amount of protein was subjected to electrophoresis on sodium dodecyl sulfate (SDS)-polyacrylamide gels. The gels were then transferred to polyvinylidene fluoride (PVDF) membranes. The blots were incubated overnight at 4 °C with the desired antibodies and then with the diluted enzyme-linked secondary antibodies and then visualized by enhanced chemiluminescence according to the recommended procedure^27^. The results are representative of three independent experiments. The enhanced chemiluminescence (ECL) detection reagents were purchased from Pierce Biotechnology (Rockford, IL, USA). Antibodies against N-cadherin, E-cadherin, p65, p-p65, TNF-α, IL-1β, COX-2, cyclin B1, cyclin D1, and GAPDH were purchased from Cell Signaling Technology (Beverly, MA, USA), and antibodies against PARP, caspase-3, BCL-2, HBsAg and HBeAg were obtained from Sigma-Aldrich. Peroxidase-labeled goat anti-rabbit and goat anti-mouse immunoglobulin were purchased from Bioworld (Minneapolis, MN, USA).

### Wound-healing assays

For the wound-healing assays, confluent monolayers of cells were lightly scratched with a 20-μL pipette tip and treated with either conditioned medium or unconditioned control medium. Images showing similar areas of the scratches were taken through a Nikon Eclipse TS 100 microscope immediately (0 h), 12 h and 24 h after scratching. TScratch software was used to measure the open wound area, which was defined as the fraction of open wound area in the image obtained at the 12- or 24-h time point compared with that at the initial time point and is provided as a percentage^28^.

### Colony formation assay

The effects of the Qizhu decoction extracts on the proliferation of primary cancer hepatocytes were assessed through a colony formation assay. Briefly, drug-treated and control cells were cultured for 7 days at 37 °C and 5% CO_2_ in DMEM supplemented with 10% fetal bovine serum, 100 mg/L streptomycin and 0.1 U/L ampicillin. The resulting colonies were stained with 0.1% crystal violet, and the numbers of colonies were then calculated.

### Cell proliferation assay

The proliferation rate of primary liver hepatocytes was determined at the indicated times through Cell Counting Kit 8 (CCK-8) assays (Sigma-Aldrich) according to the manufacturer’s recommended protocol.

### Cell cycle analysis and cell apoptosis

A flow cytometry analysis of the cellular DNA content was performed for assessment of the cell cycle phase distribution. Cells were trypsinized, washed with 1× PBS, fixed in ice-cold 70% ethanol for 1 h and stained with 50 μg/mL propidium iodide (PI) for fluorescence activated cell sorter (FACS) analysis. We analyzed the cells using a BD FACScan flow cytometer and Cell Quest acquisition and analysis software (Becton Dickinson Biosciences, Franklin Lakes, NJ, USA). Cells were also stained with the Annexin V/PI Apoptosis Detection kit (BD Biosciences) according to the manufacturer’s instructions, and the apoptosis rates of the samples were analyzed using a BD FACScan flow cytometer.

### Statistical analysis

All the results were analyzed using SPSS 17.0 (IBM, Armonk, NY, USA). The data are presented as the means ± standard deviations (SDs) from three independent experiments. Comparisons between groups were conducted using Student’s t-test. P < 0.05 was assumed to indicate a statistically significant difference.

## Results

### The Qizhu decoction extract QC inhibits DEN-induced hepatitis by suppressing NF-κB in mice

**Figure 1:**
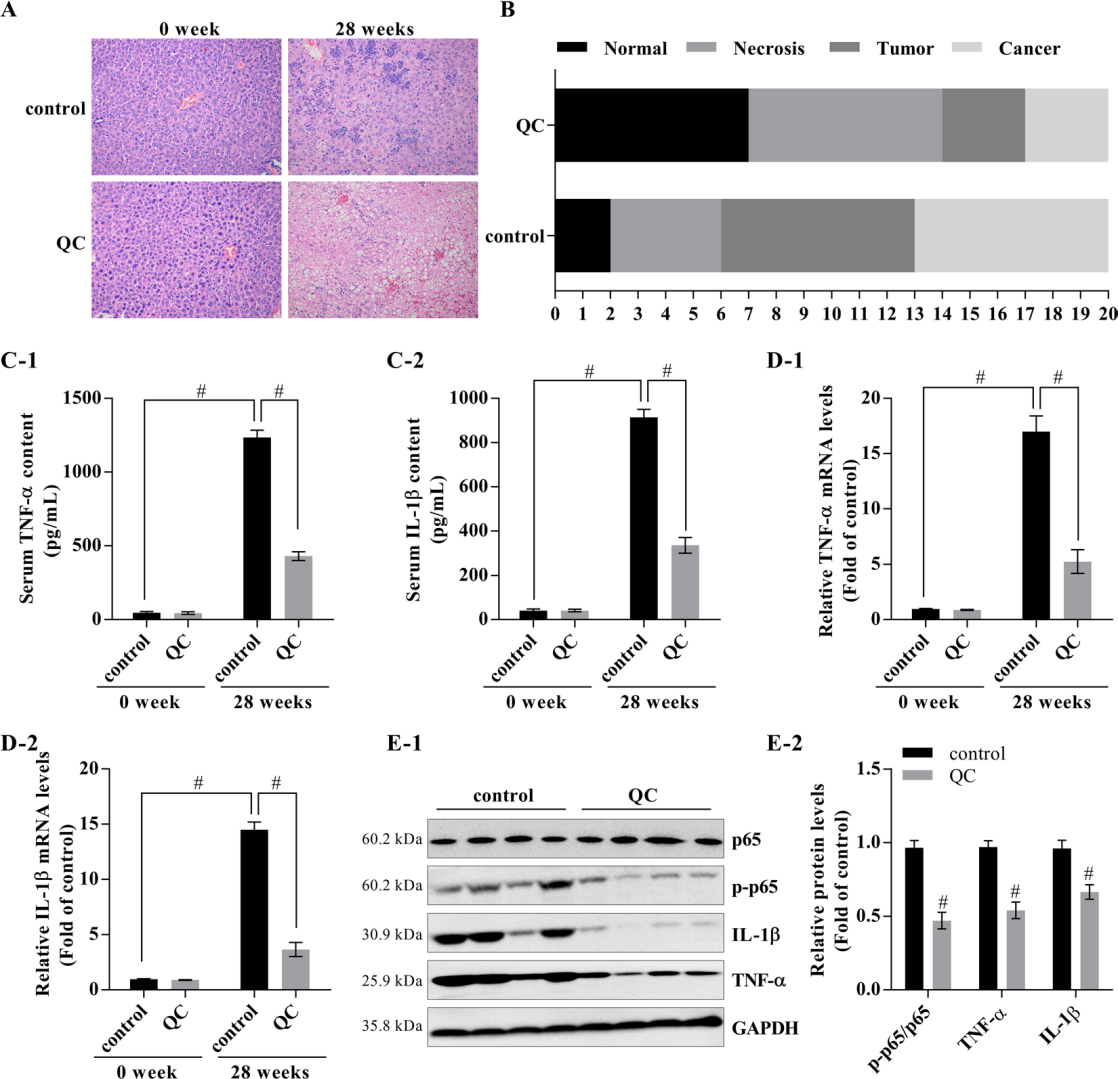
Inhibitory effect of the Qizhu decoction extracts on DEN-induced hepatitis in mice via NF-κB. Mice were given daily injections of DEN (50 mg/kg i.p.) for 28 weeks; some of the mice were only administered DEN (control), whereas others were also intragastrically administered a Qizhu decoction extract (QC) at 20 g/kg of body weight daily throughout the experimental period. (**A**) Effect of QC on DEN-induced hepatitis detected by H&E staining. (**B**) Numbers of mice with DEN-induced hepatitis, necrosis, tumor, and liver cancer. (**C**) The levels of TNF-α (C-1) and IL-1β (C-2) in mouse serum were analyzed by ELISA. Each value indicates the mean ± SD and is representative of results from three independent experiments; ^#^P < 0.001 vs. the control (0 QC). (**D**) The mRNA expression of TNF-α (D-1) and IL-1β (D-2) in mouse livers was determined by real-time PCR, and GAPDH was used as an internal control. Each value indicates the mean ± SD and is representative of results from three independent experiments; ^#^P < 0.001 vs. the control (0 QC). (**E**) The NF-κB activity was analyzed by western blot (E-1), and a corresponding semiquantitative analysis based on optical density was performed using ImageJ software (E-2). The values are presented as the means ± SDs of data from three separate experiments; ^#^P < 0.001 vs. the control (0 QC).

The mice were given daily i.p. injections of 50 mg/kg DEN (i.p.) alone or with QC for 28 weeks. This study revealed that QC can suppress DEN-induced hepatitis (Fig. 1A,B). Specifically, 35% of the mice coadministered QC with DEN exhibited a healthy status, whereas only 10% of the mice in the group that received DEN alone were healthy. In addition, QC was able to significantly downregulate the levels of TNF-α and IL-1β in mouse sera, as determined by ELISA (Fig. 1C), and a real-time PCR assay showed that QC inhibited the mRNA expression of TNF-α and IL-1β in the mouse livers (Fig. 1D). Furthermore, QC significantly repressed the protein expression levels of TNF-α, IL-1β, and p-p65, which led to inactivation of the NF-κB pathway in the livers of QC-treated mice (Fig. 1E).

### The Qizhu decoction extracts inhibit the migration and proliferation of primary cancer hepatocytes from DEN-induced HCC

**Figure 2:**
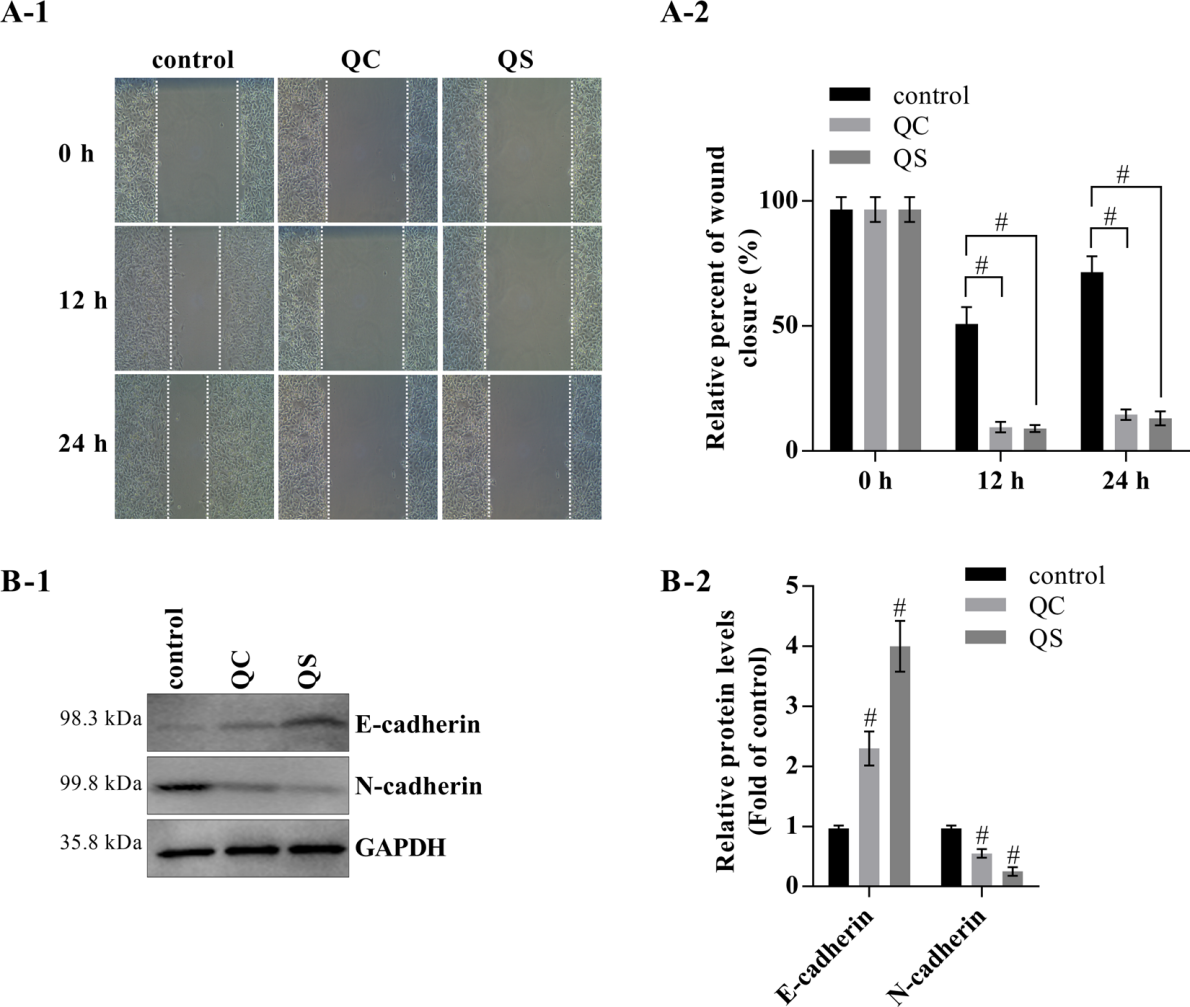
The Qizhu decoction extracts inhibit the migration of primary cancer hepatocytes in DEN-induced HCC. Mice were intragastrically administered DEN (50 mg/kg i.p.) daily for 28 weeks, and primary cancer hepatocytes were then isolated for further experiments. (**A**) Wound-healing assays with 10 μg/mL QC and QS. The migration of the cells to the wound was visualized at 0, 12, and 24 h with a Leica inverted phase-contrast microscope (×200 magnification); ^#^P < 0.001 vs. the negative control. (**B**) The levels of E-cadherin, N-cadherin, and GAPDH proteins in primary cancer hepatocytes were analyzed by western blot analysis (B-1), and a corresponding semiquantitative analysis based on optical density was performed using ImageJ software (B-2). ^#^P < 0.001 vs. the control.

The effects of the Qizhu decoction extracts (QC and QS) on the migration of primary cancer hepatocytes in DEN-induced HCC were investigated through a wound-healing assay. Photomicrographs were obtained 0 h, 12 h and 24 h after the cell monolayers were scratched, and both the QC- and QS-treated cells showed a significant decrease in migration compared with the control cells (Fig. 2A-1,A-2). Further investigation of the effects of the Qizhu decoction extracts on the regulation of biomarkers of the epithelial-to-mesenchymal transition revealed that QC and QS notably increased E-cadherin expression and reduced N-cadherin expression (Fig. 2B).

**Figure 3:**
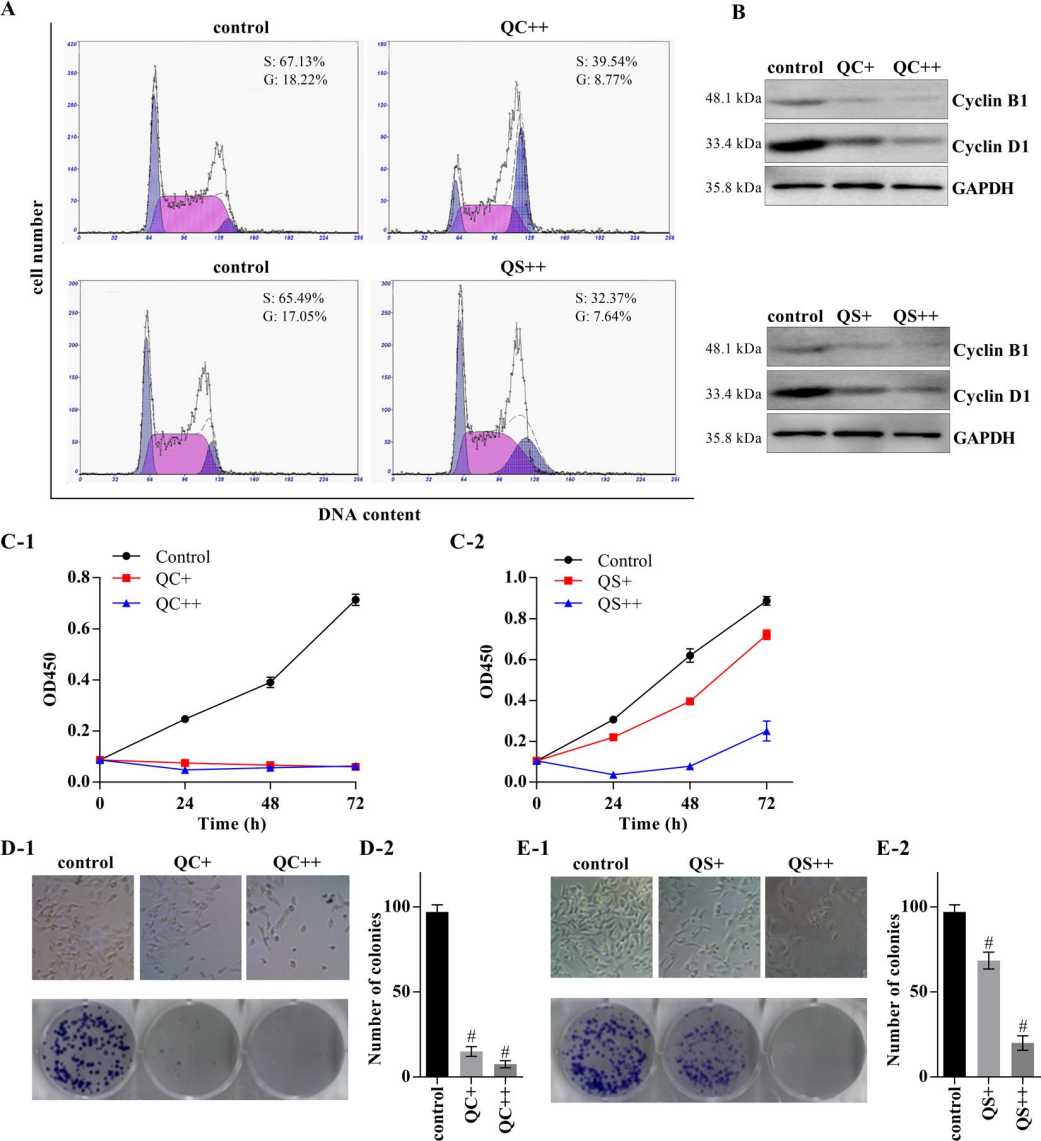
The Qizhu decoction extracts downregulate the proliferation of primary cancer hepatocytes in DEN-induced HCC. Primary cancer hepatocytes were incubated with QC and QS at concentrations of 5 μg/mL (QC+ and QS+) and 10 μg/mL (QC++ and QS++). (**A**) A flow cytometry analysis showed that 24 h of incubation with QC and QS decreased the proportions of cells in the G2 and S phases. (**B**) A western blot analysis showed that cyclin B1 and cyclin D1 expression was downregulated in the cells after 24 h of incubation with QC and QS. (**C**) A CCK-8 assay indicated that QC and QS significantly reduced cell proliferation; ^#^P < 0.001 vs. the control. (**D**,**E**) Twenty-four hours of incubation with QC (**D**) and QS (**E**) inhibited cell growth, as determined by a colony formation assay; ^#^P < 0.001 vs. the control.

We then explored the inhibitory effects of Qizhu decoction on the proliferation of primary cancer hepatocytes through flow cytometry. As shown in Fig. 3A, the data revealed that both QC and QS decreased the numbers of cancer hepatocytes from DEN-induced HCC in the G2 and S phases compared with those from the control group that did not receive QC or QS. The QC- and QS-treated cells exhibited downregulation of cyclin B1 and cyclin D1 (Fig. 3B). CCK-8 assays also demonstrated that the proliferation of cancer cells was significantly decreased after treatment with QC and QS (Fig. 3C). Moreover, a colony formation assay showed that both QC and QS significantly inhibited the proliferation of cancer cells in a dose-dependent manner (Fig. 3D,E).

### The Qizhu decoction extracts induce the apoptosis of primary cancer hepatocytes from DEN-induced HCC

**Figure 4:**
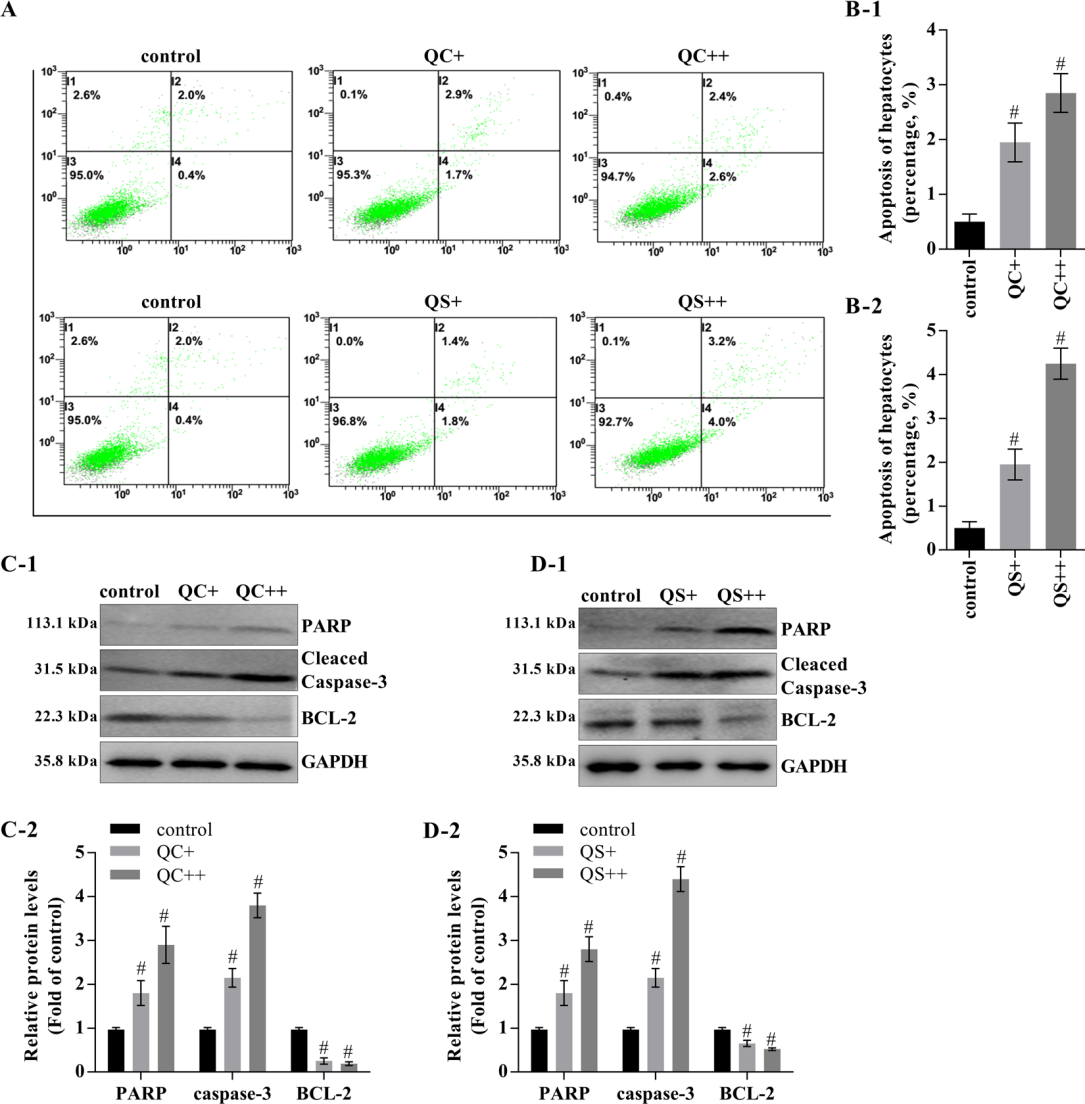
The Qizhu decoction extracts promote apoptosis of primary cancer hepatocytes in DEN-induced HCC. Primary cancer hepatocytes were isolated from the livers of mice with DEN-induced HCC and incubated for 24 h with QC and QS at concentrations of 5 μg/mL (QC+ and QS+) and 10 μg/mL (QC++ and QS++). (**A**,**B**) Representative flow cytometry analysis of apoptosis. The cells were stained with annexin V and PI (**A**). The data shown are the means ± SDs of three independent experiments; ^#^P < 0.001 vs. the control (**B**). (**C**,**D**) Representative western blots with the indicated antibodies (C-1 and D-1). A corresponding semiquantitative analysis based on optical density was performed using ImageJ software (C-2 and D-2). GAPDH was used as a normalization control; ^#^P < 0.001 vs. the control.

We investigated whether QC and QS regulated apoptosis in primary cancer hepatocytes and found that the cells incubated with QC and QS exhibited higher apoptosis percentages (2.6% and 4%, respectively) than those that were not incubated with either extract (0.4%) (Fig. 4A,B). Notably, the Qizhu decoction extracts increased the expression of cleaved caspase-3 and PARP and reduced BCL-2 expression, which suggested that BCL-2 is critical for the ability of the Qizhu decoction extracts to modulate apoptosis (Fig. 4C and C). Taken together, the evidence shows that both Qizhu decoction extracts (QC and QS) inhibit the migration and proliferation of primary cancer hepatocytes in DEN-induced HCC.

### The Qizhu decoction extracts inhibit HCC, and this effect is dependent on the NF-κB pathway

**Figure 5:**
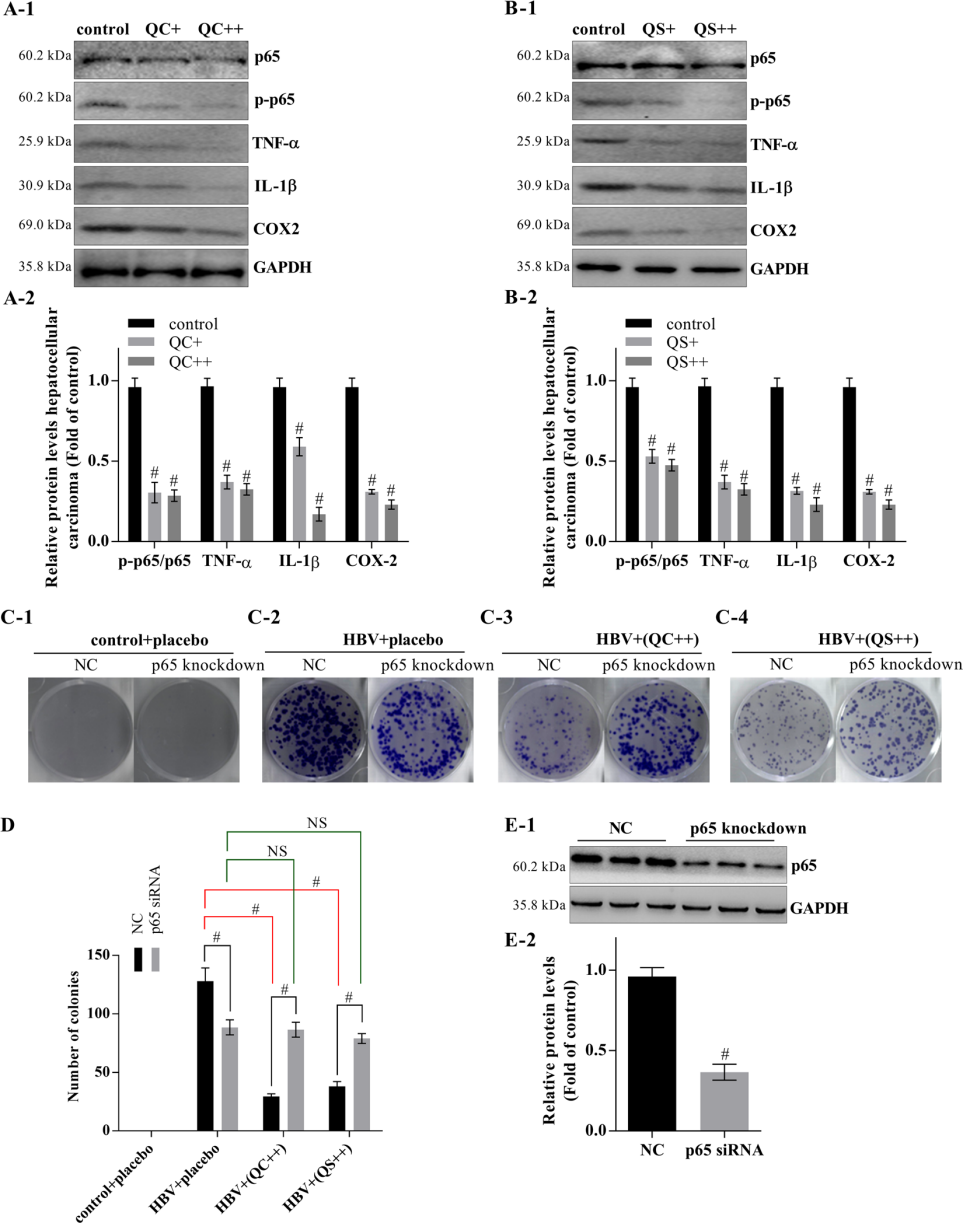
The Qizhu decoction extracts depend on NF-κB to inhibit DEN-induced HCC. The primary cancer hepatocytes were incubated with QC and QS for 24 h at concentrations of 5 μg/mL (QC+ and QS+) and 10 μg/mL (QC++ and QS++). (**A**,**B**) Effects of QC and QS on the protein levels of p65, p-p65, TNF-α, IL-1β, and COX-2 analyzed by real-time PCR. GAPDH was used as an internal control. Each value indicates the mean ± SD and is representative of results from three independent experiments; ^#^P < 0.001 vs. control. (**C**) QC and QS inhibited cell growth dependent on NF-κB, as determined by a colony formation assay. (**D**) The numbers of colonies were calculated; ^#^P < 0.001 vs. control. (**E**) Knockdown of p65 in liver cancer cells of mice analyzed by western blot.

Both QC and QS significantly inhibited the phosphorylation of p65, resulting in the inhibition of NF-κB signaling (Fig. 5A,B). In addition, QC and QS reduced the colony counts by 22.67% and 29.69%, respectively (Fig. 5C). However, neither QC nor QS inhibited the proliferation of primary cancer hepatocytes in which p65 was knocked down (Fig. 5C–E).

### The Qizhu decoction extracts inhibit HBV DNA synthesis and the secretion of HBsAg and HBeAg by suppressing NF-κB

**Figure 6:**
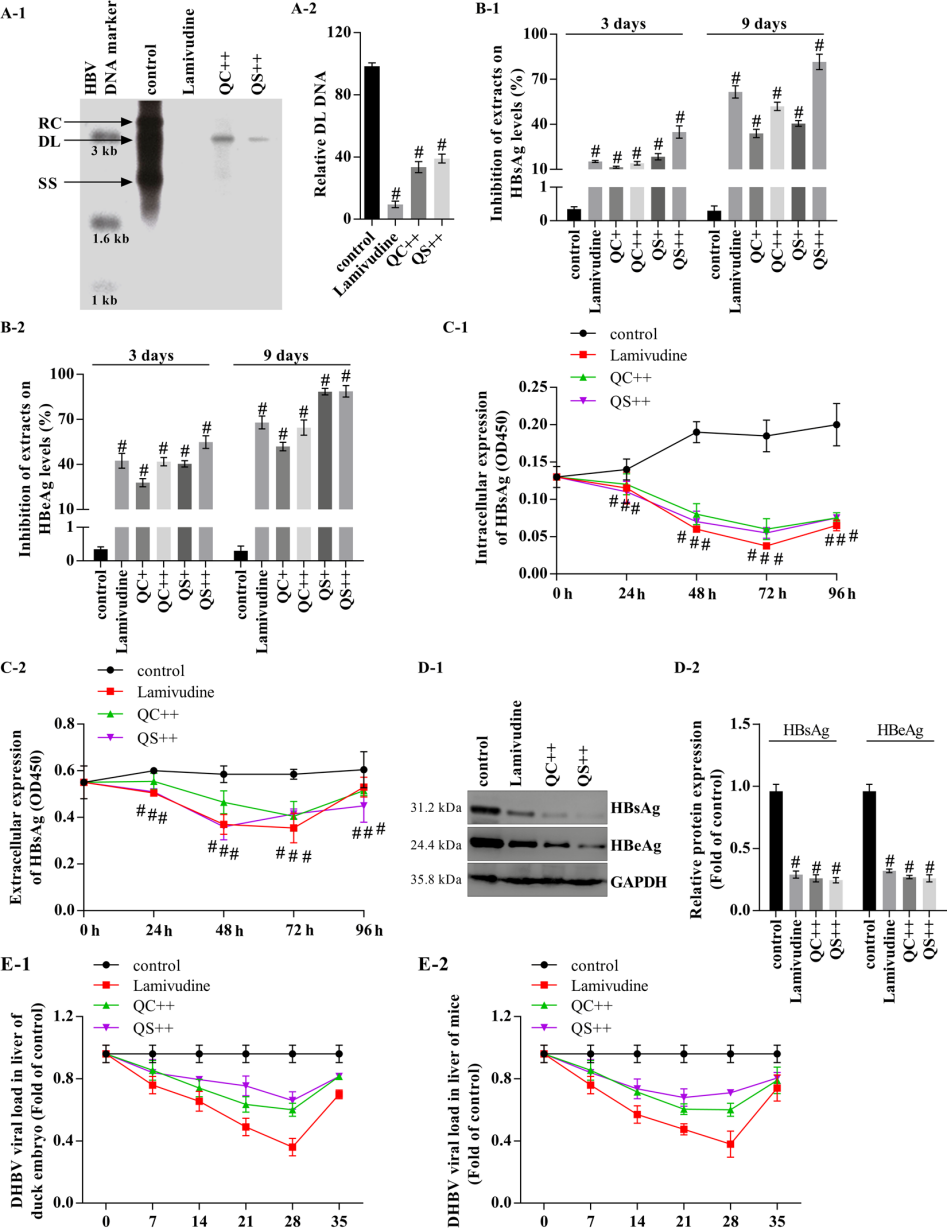
Qizhu decoction extracts inhibit HBV DNA synthesis and the secretion of HBsAg and HBeAg *in vitro* and *in vivo*. (**A**) Southern blotting to determine HBV DNA synthesis after treatment with lamivudine or QC and QS. At 1 day after transfection with HBV DNA, HepG2 cells were treated with QC and QS at concentrations of 5 μg/mL (QC+ and QS+) and 10 μg/mL (QC++ and QS++) or with lamivudine (4 μg/mL) for 48 h. HBV DNA was then extracted from isolated core particles, separated, transferred to nylon membranes, hybridized with a random-primed ^32^P-labeled HBV-specific probe, and subjected to autoradiography. Single-stranded and double-stranded linear DNA and partially double-stranded relaxed circular DNA are marked as SS, DL, and RC, respectively (A-1). The graph shows the level of DL DNA relative to that of HBV DNA in the absence of treatment, as measured by a Tanon 6600 Luminescent Imaging Workstation (A-2). Each value indicates the mean ± SD and is representative of results from three independent experiments; ^#^P < 0.001 vs. control. (**B**) QC and QS inhibited the secretion of HBsAg and HBeAg from HBV WT DNA-transfected HepG2 cells (determined by ELISA). Each value indicates the mean ± SD and is representative of results from three independent experiments; ^#^P < 0.001 vs. control. (**C**) QC and QS inhibited intracellular and extracellular HBsAg in HBV WT DNA-transfected PLC/PRF/5 cells (determined by ELISA). Each value indicates the mean ± SD and is representative of results from three independent experiments; ^#^P < 0.001 vs. control. (**D**) QC and QS repressed intracellular HBsAg and HBeAg expression in HepG2 cells (determined by western blot). ^#^P < 0.001 compared with the control. Each value indicates the mean ± SD and is representative of results from three independent experiments; ^#^P < 0.001 vs. control. (**E**) Inhibitory effects of 30 g QC and QS/kg body weight daily on HBV DNA synthesis analyzed by real-time PCR in Guangzhou brown spotted ducks with congenital and acquired duck hepatitis B virus infection (E-1) and on HBV-induced hepatitis in mice (E-2). Lamivudine was used as a positive control at 200 mg/kg body weight daily.

We examined the inhibitory effect of Qizhu decoction on HBV DNA synthesis. Specifically, four days after treatment, the levels of all viral replicative intermediates in HepG2 cells were significantly reduced in the groups treated with 5 μg/mL QC and QS compared with the levels in the untreated group (Fig. 6A). The administration of 6 μg/mL QC and QS exerted similar repressive effects on the HBV DNA levels (Fig. 6A). HepG2 cells and PLC/PRF/5 cells were harvested after treatment, and their HBsAg and HBeAg levels on days 3, 4 and 9 after drug application were examined by ELISA. The results showed that the Qizhu decoction extracts inhibited the intracellular expression of HBsAg and HBeAg (Fig. 6B,C). Specifically, the HBsAg and HBeAg levels in the QC- and QS-incubated groups were significantly lower than those of the virus group. Subsequently, a western blotting analysis showed that QC and QS significantly inhibited HBsAg and HBeAg expression in HepG2 cells by approximately 68% and 73%, respectively (Fig. 6D-1,D-2).

**Figure 7:**
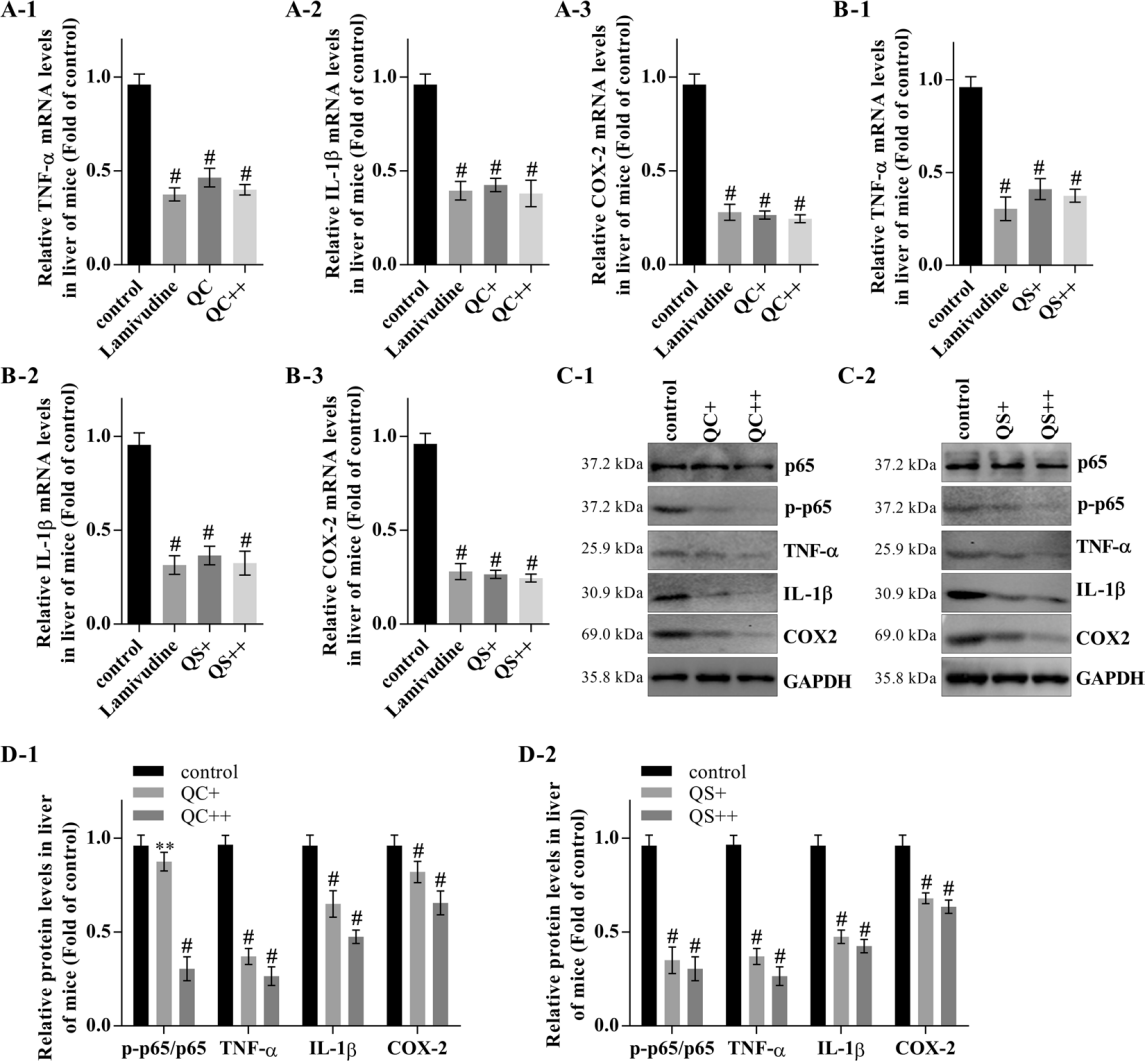
Inhibitory effect of Qizhu decoction extracts on HBV-induced hepatitis in mice involves the suppression of NF-κB. (**A**) The effect of QC on the mRNA expression of TNF-α, IL-1β, and COX-2 in mouse livers was analyzed by real-time PCR. GAPDH was used as an internal control. Each value indicates the mean ± SD and is representative of results from three independent experiments; ^#^P < 0.001 vs. the control. (**B**) The effect of QS on the mRNA expression of TNF-α, IL-1β, and COX-2 in mouse livers was analyzed by real-time PCR. GAPDH was used as an internal control. Each value indicates the mean ± SD and is representative of results from three independent experiments; ^#^P < 0.001 vs. the control. (**C**,**D**) NF-κB activity in mouse livers was analyzed by western blot (**C**), and a corresponding semiquantitative analysis based on optical density was performed using ImageJ software (**D**). The values are presented as the means ± SDs of data from three separate experiments; ^#^P < 0.001 vs. the control.

QC and QS also inhibited HBV DNA synthesis *in vivo* in Guangzhou brown spotted ducks with congenital and acquired duck hepatitis B virus infection (Fig. 6E-1) and in mice with HBV-induced hepatitis (Fig. 6E-2). A real-time PCR analysis showed that QC and QS inhibited the mRNA expression of TNF-α and IL-1β in a dose-dependent manner in the mouse livers (Fig. 7A,B). Furthermore, QC and QS repressed the protein expression of TNF-α, IL-1β, and p-p65, leading to inactivation of the NF-κB pathway (Fig. 7C,D).

### Analysis of the main compounds of Qizhu decoction by liquid chromatography and mass spectrometry

**Figure 8:**
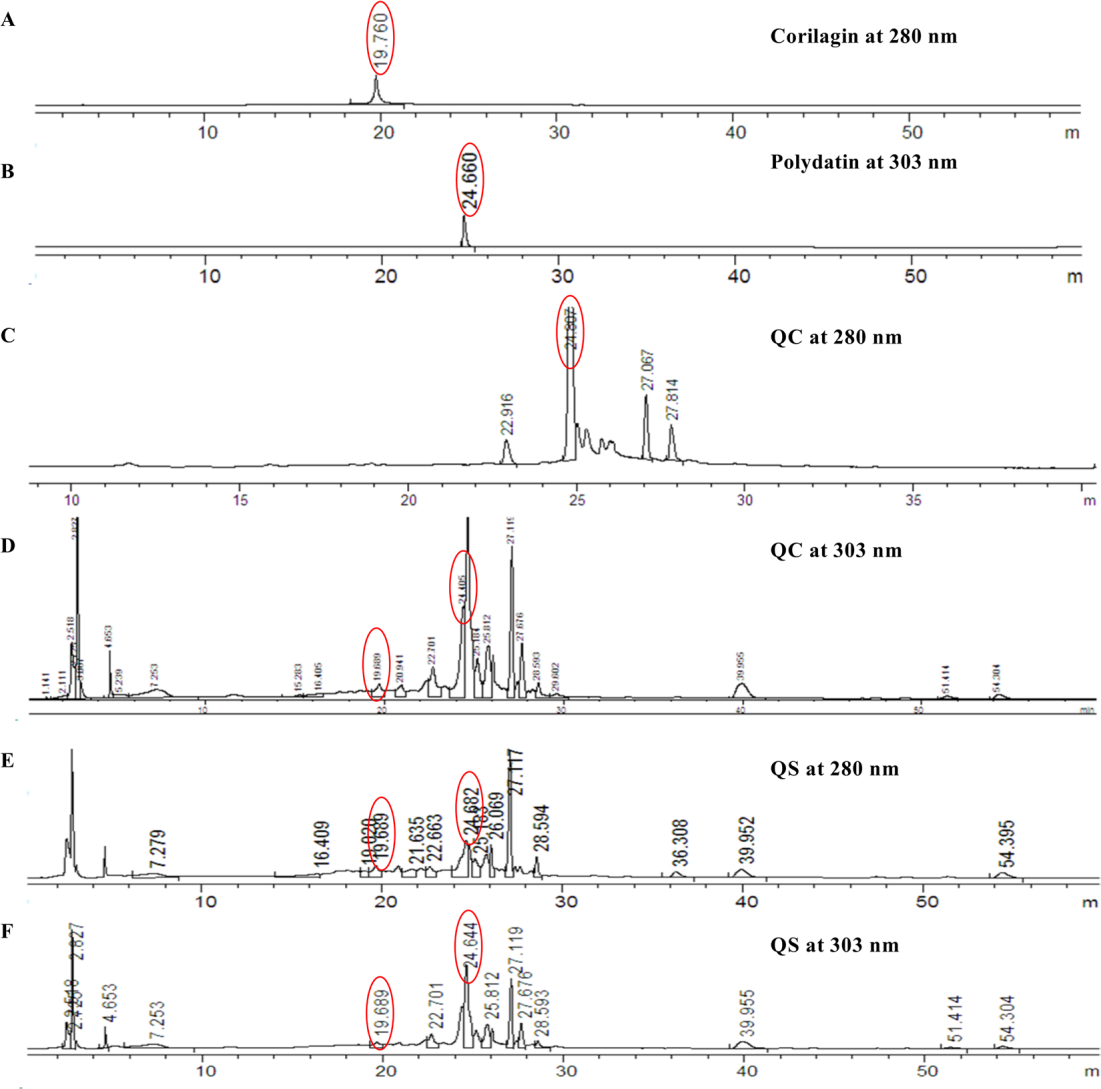
Liquid chromatography/quadrupole time-of-flight mass spectrometry analysis of QC and QS from Qizhu decoction. (**A**) Corilagin (13%) at 280 nm. (**B**) Polydatin (6%) at 303 nm. (**C**) QC (12%) at 208 nm. (**D**) QC (12%) at 303 nm. (**E**) QS (11%) at 208 nm. (**F**) QS (10%) at 303 nm.

It has been reported that corilagin and polydatin exhibit anticancer and anti-inflammatory activity and might be useful for hepatoprotection^18,19,21,29–32^. In this study, corilagin and polydatin were detected in the Qizhu decoction extracts (in both QC and QS), and the corilagin (1.14 mg/mL) and polydatin (0.29 mg/mL) standards were detected as major peaks (Fig. 8A,B). Thus, corilagin and polydatin might be the compounds responsible for the effects of QC (Fig. 8C,D) and QS (Fig. 8E,F). However, these compounds need to be tested, and the other major peaks shown in Fig. 8 need to be identified in future studies.

## Discussion

NF-κB plays critical roles in spontaneous liver injury, chronic inflammation, fibrosis, and HCC^13–15^. Here, we found that extracts (QC and QS) of Qizhu decoction can significantly inhibit DEN- or HBV-induced inflammation and liver cancer by suppressing NF-κB signaling *in vitro* and in C57BL/6 mice. After the disruption of NF-κB signaling by gene knockdown, the isolated primary cancer hepatocytes of mice formed decreased numbers of colonies *in vitro*. QC and QS inhibited HBV DNA replication, and corilagin and polydatin were identified by liquid chromatography and mass spectrometry as components of both extracts. Thus, these compounds might be key components of QC and QS.

As a traditional Chinese medicine, Qizhu decoction, which contains *Phyllanthus urinaria*, *Polygonum cuspidatum*, *Hedyotis diffusa*, *Paeonia lactiflora*, *Paeonia veitchii*, *Rhizoma Smilacis Glabrae*, and *Salvia miltiorrhiza*^16^, has been widely used in China. Several compounds that have been isolated from *P. cuspidatum* and *P. urinaria*, such as corilagin, polydatin, resveratrol, quercetin, emodin, and rutin, among others, exert significant hepatoprotective effects against CCl_4_-induced acute liver injury and HBV-induced hepatitis in mice, and the underlying mechanisms might be related to their antioxidant and anti-inflammatory effects^18,19,21,29,31,32^. These compounds also exhibit anticancer activity^29,30^. Therefore, corilagin and polydatin might represent the active ingredients of the Qizhu decoction extracts (QC and QS) and might thus be valuable for the treatment of hepatitis and even liver cancer. In fact, we determined the precise components of Qizhu decoction in another study, which was published in Chinese (Chin Hosp Pharm J, 2011 Sep, Vol 13, NO.18.), and we are currently conducting studies on the effects of the active components of the decoction on hepatitis and hepatocellular carcinoma *in vitro* and in C57BL/6 mice and will publish the data in a future article.

We first explored the inhibitory effect of QC on DEN-induced hepatitis and HCC. Our *in vivo* data showed that QC decreased the prevalence of DEN-induced hepatitis and HCC in mice, which indicated that QC might prevent the progression of hepatitis to HCC. We also found that QC reduced the levels of TNF-α and IL-1β in the serum and livers of mice. It has been demonstrated that corilagin and polydatin inhibit NF-κB activation^33–35^, and in agreement with this finding, QC inhibited the phosphorylation of p65 and thus inactivated the NF-κB pathway in mice with DEN-induced liver cancer. All these data indicate that NF-κB signaling is involved in the mechanism underlying the effects of QC in preventing or reducing the prevalence of hepatitis and hepatic cancers in mice. NF-κB signaling plays critical roles in not only in liver injury, chronic inflammation, and fibrosis but also HCC^1^. It is known that the knockdown of NF-κB regulators leads to hepatitis and HCC. Thus, we can reasonably propose that Qizhu decoction might be used for the treatment of HCC^14,15^.

As expected, the data showed that QC and QS inhibited the migration of mouse primary cancer hepatocytes by upregulating E-cadherin, which promotes the adhesion of cancer cells^36^. A flow cytometry analysis showed that QC and QS acted as important cell cycle regulators during the G1/S phase. In addition, QC and QS reduced the colony formation ability of cells in a dose-dependent manner. Our analysis of the regulatory mechanisms of QC and QS on the cell cycle revealed that QC and QS inhibited the expression of cyclin D1 and cyclin B1, which suggests that the two extracts can regulate steps in the cell cycle.

The numbers of apoptotic cells among the QC- and QS-treated primary cancer hepatocytes was increased compared with that found among nontreated controls. We also observed a significant increase in the level of cleaved caspase-3, and this effect was accompanied by a decrease in BCL-2 expression. Taken together, these findings indicate that QC and QS might be activators of liver cancer cell apoptosis. Additionally, we found that QC and QS decreased the expression of TNF-α and IL-1β by inactivating NF-κB signaling. Importantly, QC and QS did not significantly inhibit the growth (or colony formation) of primary cancer hepatocytes after p65 knockdown. Consequently, these findings suggest that the inhibitory effects of QC and QS in liver cancer are linked to the regulation of NF-κB signaling. It is known that polydatin inhibits cell proliferation and induces apoptosis in laryngeal cancer and HeLa cells through suppression of the PDGF/AKT signaling pathway^37^. Corilagin promotes apoptosis in SGC-7901 human gastric cancer cells via the mitochondrial pathway and inhibits HCC cell proliferation by inducing G2/M phase arrest^38,39^. Because both of these compounds are present in QC and QS, polydatin and corilagin might be the active chemicals of these extracts and likely perform their anticancer activity by repressing NF-κB activation. Further studies are needed to investigate the difference between the effects of QS and QC in the treatment of hepatitis and hepatocellular carcinoma.

HBV infection is a global health concern because the infected individuals are at a high risk for developing liver cirrhosis and eventually HCC^9–12^. The data obtained in this study indicate that QC and QS significantly suppressed HBV replication and the expression of HBsAg and HBeAg in an HBV transfection model *in vitro* and in mice and Guangzhou brown spotted ducks *in vivo*. Furthermore, QC and QS reduced the expression of TNF-α and IL-1β by inactivating NF-κB signaling in the livers of HBV-infected mice. Specifically, the experimental data showed that corilagin and polydatin depressed HBV replication and HBV-induced hepatitis. Taken together, the evidence obtained in this study show that QC and QS, as well as corilagin and polydatin, showed activity against HBV-induced hepatitis, possibly through the NF-κB signaling pathway.

In conclusion, QC and QS might show activity against DEN- and HBV-induced hepatitis and HCC and might exert this effect by regulating the NF-κB pathway *in vitro* and in C57BL/6 mice.

## Data Availability

All data generated or analyzed during this study are included in this published article (and its Supplementary Information files).

## References

[1] Luedde T, Schwabe RF (2011). NF-kappa B in the liver-linking injury, fibrosis and hepatocellular carcinoma. Nat Rev Gastro Hepat.

[2] Rajewsky MF, Dauber W, Frankenberg H (1966). Liver Carcinogenesis by Diethylnitrosamine in Rat. Science.

[3] Umeda T, Hino O (2002). Molecular aspects of human hepatocarcinogenesis mediated by inflammation: From hypercarcinogenic state to normo- or hypocarcinogenic state. Oncology-Basel.

[4] Mantovani A (2005). Cancer - Inflammation by remote control. Nature.

[5] Liu YF (2009). Characteristic gene expression profiles in the progression from liver cirrhosis to carcinoma induced by diethylnitrosamine in a rat model. J Exp Clin Canc Res.

[6] Torre LA (2015). Global Cancer Statistics, 2012. Ca-Cancer J Clin.

[7] Llovet, J. M. *et al*. Hepatocellular carcinoma. *Nat Rev Dis Primers* **2**, Artn 16018, 10.1038/Nrdp.2016.18 (2016). 10.1038/nrdp.2016.18 27158749

[8] El-Serag HB (2012). Epidemiology of Viral Hepatitis and Hepatocellular Carcinoma. Gastroenterology.

[9] El-Serag HB (2012). Epidemiology of viral hepatitis and hepatocellular carcinoma (vol 142, pg 1264, 2012). Gastroenterology.

[10] Fattovich G (2008). Long-term outcome of chronic hepatitis B in Caucasian patients: mortality after 25 years. Gut.

[11] Chen CJ, Yang HI (2011). Natural history of chronic hepatitis B REVEALed. J Gastroen Hepatol.

[12] Kim BK, Han KH, Ahn SH (2011). Prevention of Hepatocellular Carcinoma in Patients with Chronic Hepatitis B Virus Infection. Oncology-Basel.

[13] Mah, W. C. *et al*. Methylation Profiles Reveal Distinct Subgroup of Hepatocellular Carcinoma Patients with Poor Prognosis (vol 9, e104158, 2014). *Plos One* **11**, ARTN e0146690, 10.1371/journal.pone.0146690 (2016). 10.1371/journal.pone.0104158 PMC4122406 25093504

[14] Bettermann K (2010). Tak1 Suppresses a Nemo-Dependent, but Nf-Kappab-Independent Pathway to Liver Cancer. J Hepatol.

[15] Inokuchi S (2010). Disruption of TAK1 in hepatocytes causes hepatic injury, inflammation, fibrosis, and carcinogenesis. P Natl Acad Sci USA.

[16] Guo Q (2017). Liver metabolomics study reveals protective function of Phyllanthus urinaria against CCl4-induced liver injury. Chin J Nat Medicines.

[17] Wang XJ, Ichikawa H, Konishi T (2001). Antioxidant potential of Qizhu Tang, a Chinese herbal medicine, and the effect on cerebral oxidative damage after ischemia reperfusion in rats. Biol Pharm Bull.

[18] Jung, J. *et al*. Inhibitory effect of Phyllanthus urinaria L. extract on the replication of lamivudine-resistant hepatitis B virus *in vitro*. *Bmc Complem Altern M* **15**, Artn 255, 10.1186/S12906-015-0792-3 (2015). 10.1186/s12906-015-0792-3 PMC4518506 26220282

[19] Wu Y (2015). Extract from Phyllanthus urinaria L. inhibits hepatitis B virus replication and expression in hepatitis B virus transfection model *in vitro*. Chin J Integr Med.

[20] Park, S., Lim, J., Kim, J. R. & Cho, S. Inhibitory effects of resveratrol on hepatitis B virus X-protein (HBx)-induced hepatocellular carcinoma (HCC). *Faseb Journal* **30** (2016). 10.4142/jvs.2017.18.4.419 PMC5746434 28385009

[21] Yang F (2016). Effect of Corilagin on the miR-21/smad7/ERK signaling pathway in a schistosomiasis-induced hepatic fibrosis mouse model. Parasitol Int.

[22] Dang, S. S. *et al*. Inhibition of the replication of hepatitis B virus *in vitro* by emodin. *Med Sci Monitor* **12**, Br302-Br306 (2006). 16940925

[23] Guidotti LG, Matzke B, Schaller H, Chisari FV (1995). High-Level Hepatitis-B Virus-Replication in Transgenic Mice. J Virol.

[24] Wang J (2010). CD137-Mediated Pathogenesis from Chronic Hepatitis to Hepatocellular Carcinoma in Hepatitis B Virus-Transgenic Mice. J Immunol.

[25] Klaunig JE (1981). Mouse liver cell culture. I. Hepatocyte isolation. In Vitro.

[26] Aktan F (2006). Gingerol metabolite and a synthetic analogue Capsarol inhibit macrophage NF-kappaB-mediated iNOS gene expression and enzyme activity. Planta medica.

[27] Lu, F. I., Sun, Y. H., Wei, C. Y., Thisse, C. & Thisse, B. Tissue-specific derepression of TCF/LEF controls the activity of the Wnt/beta-catenin pathway. *Nat Commu*n 5, Artn 5368, 10.1038/Ncomms6368 (2014). 10.1038/ncomms6368 25371059

[28] Geback T, Schulz MMP, Koumoutsakos P, Detmar M (2009). TScratch: a novel and simple software tool for automated analysis of monolayer wound healing assays. Biotechniques.

[29] Peng W, Qin RX, Li XL, Zhou H (2013). Botany, phytochemistry, pharmacology, and potential application of Polygonum cuspidatum Sieb.et Zucc.: A review. J Ethnopharmacol.

[30] Chudapongse N, Kamkhunthod M, Poompachee K (2010). Effects of Phyllanthus urinaria extract on HepG2 cell viability and oxidative phosphorylation by isolated rat liver mitochondria. J Ethnopharmacol.

[31] Zhang, H. *et al*. Protective Effects of Polydatin from Polygonum cuspidatum against Carbon Tetrachloride-Induced Liver Injury in Mice. *Plos One* **7**, ARTN e46574 10.1371/journal.pone.0046574 (2012). 10.1371/journal.pone.0046574 PMC3461010 23029551

[32] Du P (2016). Mechanism of Corilagin interference with IL-13/STAT6 signaling pathways in hepatic alternative activation macrophages in schistosomiasis-induced liver fibrosis in mouse model. Eur J Pharmacol.

[33] Dong XR (2010). Corilagin inhibits the double strand break-triggered NF-kappa B pathway in irradiated microglial cells. Int J Mol Med.

[34] Gambari R (2012). Corilagin is a potent inhibitor of NF-kappaB activity and downregulates TNF-alpha induced expression of IL-8 gene in cystic fibrosis IB3-1 cells. Int Immunopharmacol.

[35] Ye, J. *et al*. Polydatin inhibits mast cell-mediated allergic inflammation by targeting PI3K/Akt, MAPK, NF-kappa B and Nrf2/HO-1pathways. *Sci Rep-Uk* **7**, Artn 11895, 10.1038/S41598-017-12252-3 (2017). 10.1038/s41598-017-12252-3 PMC5605538 28928455

[36] Pan YY (2016). The Critical Role of Rab31 in Cell Proliferation and Apoptosis in Cancer Progression. Mol Neurobiol.

[37] Li, H. X., Shi, B. Y., Li, Y. Y. & Yin, F. F. Polydatin inhibits cell proliferation and induces apoptosis in laryngeal cancer and HeLa cells via suppression of the PDGF/AKT signaling pathway. *J Biochem Mol Toxi*c **3**1, ARTN e21900 10.1002/jbt.21900, (2017). 10.1002/jbt.21900 28266802

[38] Wang BQ (2013). Corilagin nanoparticle-induced apoptosis in human gastric cancer SGC-7901 cells via the mitochondrial pathway. Acta Pharmacol Sin.

[39] Ming YL (2013). Corilagin inhibits hepatocellular carcinoma cell proliferation by inducing G2/M phase arrest. Cell Biol Int.

